# A Great Challenge: Preserve or Improve the Classification of the Journal Investigación y Educación en Enfermería*

**DOI:** 10.17533/udea.iee.v41n3e16

**Published:** 2023-10-31

**Authors:** María del Pilar Pastor-Durango

**Affiliations:** 1 Nurse, Ph.D. Retired Professor, Faculty of Nursing, University of Antioquia, Colombia. Director of the journal Investigación y Educación en Enfermería from June 2008 to September 2009. Email: ppastordurango@gmail.com Universidad de Antioquia Faculty of Nursing University of Antioquia Colombia ppastordurango@gmail.com

The first issue of the IEE Journal was published in September 1983 and it was the beginning of a permanent growth process for the dissemination of knowledge in Nursing, product of research, theoretical reflection, and exchange of care experiences. It was regulated by Agreement 027 of 02 August of 2005 by the Academic Council of the Faculty of Nursing at Universidad de Antioquia, which defines it as “the dissemination body, through which technical and scientific knowledge is disseminated referring to health and disease and to processes related with such, to the practice of Nursing and of other health and related disciplines. It also constitutes a channel to exchange knowledge and experiences with national and foreign disciplines of the social and health sciences”. 

Since its creation, the Journal was directed by nurses of high professional, scientific, and personal quality; hence, assuming its direction was a great challenge: surpass or at least maintain the Journal's classification at the national level and enhancing its visibility among professionals from other countries. Dean Beatriz Helena Ospina Rave invited me to participate as the Journal's director by mid-2008 because its current director would step down to enjoy retirement. This offer filled me with pride, while it implied assuming a great responsibility to perform in a new position for me, where I had no experience. However, motivated by the director, Professor Bertha Ligia Diez Mejía and with her guidance and companionship, I assumed the challenge, in the midst of the Journal's 25^th^ anniversary celebration.

Bearing in mind the Action Plan and the 2007-2009 Operational Plan of the Faculty of Nursing at Universidad de Antioquia, the mission of the Journal *Investigación y Educación en Enfermería*, and the strategies proposed to achieve the goals defined, progress during this period focused on systematizing the journal and constructing a methodological memory of its operation that facilitated the induction of new people. During my direction, three issues and a supplement were published, a training process for writing scientific articles that had begun with the previous director, Professor Bertha Ligia Diez, continued in which the journals began with an article that guided future authors for good writing. The intention was that articles published in each issue would respond to similar themes and that research results from different methodological approaches would be included, with the participation from authors external to the Faculty, as a recommendation by the databases of serial scientific publications. 

There was a transition in the cover design that initially included works of art related to health, as a contribution by the University Museum, but later as a recommendation from members of the academic community and with the approval of the Editorial Board, photographs were included showing different facets of nursing care. Regarding the Journal’s systematization, progress was made in implementing the Open Journal System, a free-use tool that facilitates editing of specialized journals from the Internet; in addition to allowing them to have a website with different aids for readers.

The journal's website was updated and installed on the University’s Toné server, which had manuals for its installation, configuration, administration, and interaction with the authors, reviewers and editors. Likewise, the full text of the issues was available since 2003 and all previous issues were scanned, with some requiring revision and new scanning for entry onto the Web. 

All the information in the journal was backed up manually, at least twice a week, to avoid information loss due to misuse, technical failures, or computer viruses. The database of subscribers and readers was updated and two management systems were developed to facilitate delivery of copies (subscribers) and inventory management (DBJournal). In addition, a database was constructed to update the resumes of peer reviewers and authors. 

Establishing editorial management processes required much time and dedication, but it was necessary to standardize everything. This involved publishing the journal, facilitating the induction of those who arrived, and serving as permanent reference material for all who participated in the processes of editing, publishing, distributing, and inclusion in different databases of each journal issue, as well as coordinating with other departments of the Faculty of Nursing and Universidad de Antioquia, in addition to those responsible for each process.

The Journal was already indexed in the SciELO, LILACS, PUBLINDEX, Oceano and AcademicONFILE databases. During my period as journal Director, all information requested up to volume 27, number 1 of March 2009 was delivered for the verification process for inclusion of the Journal in the Redalyc database. Regarding the CINAHL database, the data was not updated because this required subscription payment by the University, but at the time it was not valid.

For the Journal's classification in PUBLINDEX to continue in force or increase in category, the information was updated according to the requirements by COLCIENCIAS (today Ministry of Science, Technology, and Innovation), immediately after publishing each number of the journal. However, during the short time I remained as director, I did not receive a new classification.

Marketing continued by promoting the journal in events scheduled by the Faculty, ANEC, ACOFAEN, and other associations and health institutions in which professors or students participated, and who fulfilled the role of volunteer promoters of the journal, encouraging subscription, sale or donation of some numbers from the journal and who supported the updating of the inventory. The journal was also promoted and article writing was encouraged during the induction of new students to the Faculty of Nursing at undergraduate and graduate levels, and in different research seminars.

The IEE Journal, during my period as director, had contributions and determined support from many people and to whom I express my eternal gratitude. The time I spent at the journal was full of valuable lessons, of new experiences, and much commitment to ensure that the journal had greater international visibility and more agility in the review and publication of good scientific articles; however, life had prepared new experiences and great challenges for me that I had to assume as professional and citizen commitment. 

